# Enhancement of Ferroelectricity in 5 nm Metal-Ferroelectric-Insulator Technologies by Using a Strained TiN Electrode

**DOI:** 10.3390/nano12030468

**Published:** 2022-01-29

**Authors:** Cheng-Hung Wu, Kuan-Chi Wang, Yu-Yun Wang, Chenming Hu, Chun-Jung Su, Tian-Li Wu

**Affiliations:** 1 International College of Semiconductor Technology, National Yang Ming Chiao Tung University, Hsinchu 30010, Taiwan; robin.icst08g@nctu.edu.tw (C.-H.W.); kcwang.icst09g@nctu.edu.tw (K.-C.W.); yuyunwang.c@nycu.edu.tw (Y.-Y.W.); hu@eecs.berkeley.edu (C.H.); 2 Department of Electrical Engineering and Computer Science, University of California, Berkeley, CA 94720, USA; 3 Department of Electrophysics, National Yang Ming Chiao Tung University, Hsinchu 30010, Taiwan; 4 Taiwan Semiconductor Research Institute, Hsinchu 30010, Taiwan

**Keywords:** ferroelectric, HfZrO_2_, strained TiN, compressive, sub-5 nm

## Abstract

In this work, the ferroelectric characteristic of a 5 nm Hf_0.5_Zr_0.5_O_2_ (HZO) metal-ferroelectric-insulator-semiconductor (MFIS) device is enhanced through strained complementary metal oxide semiconductor (CMOS)-compatible TiN electrode engineering. Strained TiN top-layer electrodes with different nitrogen (N) concentrations are deposited by adjusting the sputtering process conditions. The TiN electrode with 18% N exhibits a compressive characteristic, which induces tensile stress in a 5 nm HZO film. A device with 18% N in TiN shows a higher remanent polarization (2Pr) and larger capacitance value than the compared sample, indicating that the strained TiN is promising for enhancing the ferroelectricity of sub-5 nm HZO devices.

## 1. Introduction

HfO_2_-based dielectrics are promising ferroelectric materials for nonvolatile memory, negative-capacitance FETs, and neuromorphic applications because of their compatibility with complementary metal-oxide-semiconductor (CMOS) technology [1,2]. To achieve the ferroelectric property, doping and annealing are often conducted for decreasing the formation energy of the orthorhombic phase [3]. Furthermore, studies have reported that the strain exerted through the different electrodes and substrates [4,5,6,7] can be used as an alternative method of inducing the ferroelectric characteristic because the tensile stress along the in-plane direction can enhance transformation from the tetragonal phase to the orthorhombic phase. Considering the compatibility with the CMOS process, TiN is considered the most important electrode that is compatible with the CMOS process because of its chemical stability, suitability, and tunable work function [8]. However, ferroelectricity is degraded when the thickness of ferroelectric HfO_2_-based dielectrics is reduced. Furthermore, to further enhance the performance in ferroelectric FETs (FeFETs) and negative-capacitance FETs (NCFETs), it is important to enhance the ferroelectricity in metal-ferroelectric-insulator-semiconductor (MFIS) devices, which is the major foundation for high-performance FeFETs and NCFETs. Therefore, in this study, we report, to the best of our knowledge for the first time, using strained TiN as a top electrode in 5 nm Hf_0.5_Zr_0.5_O_2_ (HZO) metal-ferroelectric-insulator-semiconductor (MFIS) devices to enhance ferroelectricity, showing that remanent polarization (2Pr) and the capacitance value in the accumulation region can be increased by adjusting the nitrogen (N) content in TiN.

## 2. Materials and Methods

**Figure 1:**
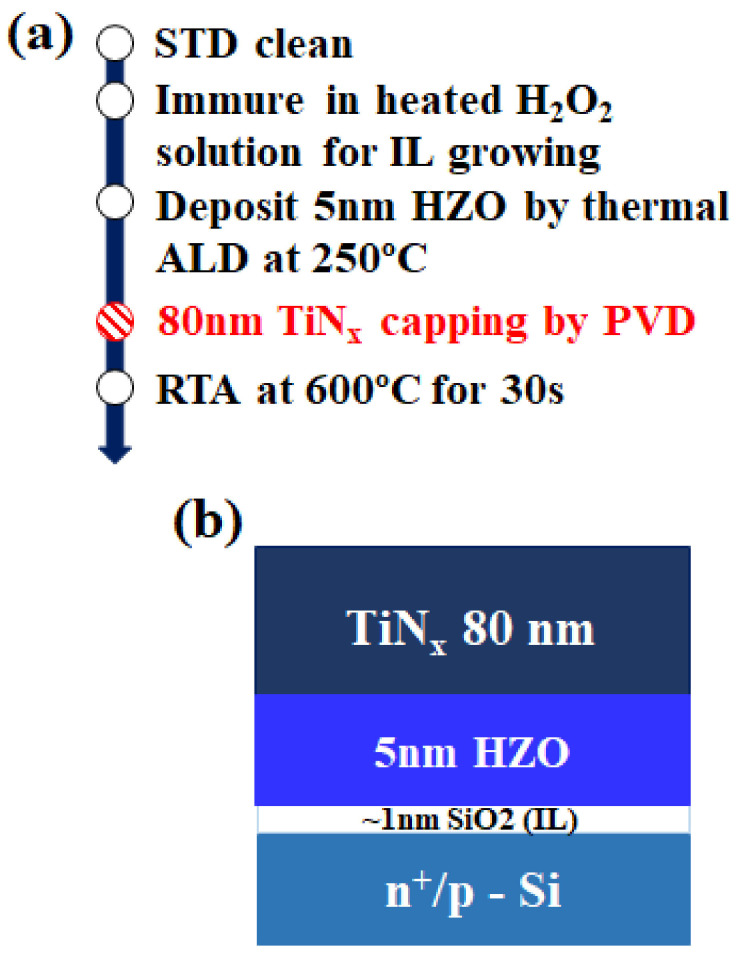
(**a**) Brief process flow for the fabrication of MFIS capacitors and (**b**) schematic of the structure of MFIS capacitors.

In this study, the devices were fabricated on a highly doped n-type substrate (<0.001 Ωcm) for P–V characterization and on a lightly doped p-type substrate (15–20 Ωcm) for capacitance voltage (C–V) measurement. The brief process flow and device schematic are depicted in Figure 1. Standard cleaning was conducted, followed by immersion of the device samples in H_2_O_2_ solution at 100 °C for 5 min to form a SiO_2_ layer with a thickness of approximately 1 nm as the interfacial layer (IL). Subsequently, 5 nm HZO was deposited using an atomic layer deposition system (PICOSUN R200 Advanced PEALD system) at 250 °C with a 1:1 cycle ratio of HfO_2_ and ZrO_2_. Tetrakis(ethylmethylamino)hafnium (TEMAH) and tetrakis(ethylmethylamino)zirconium (TEMAZ) are used as the precursors for HfO_2_ and ZrO_2_, respectively. Each cycle of HfO_2_ or ZrO_2_ is 0.83 Å, for a total of 30 cycles. The 80 nm TiN was deposited by using DC sputtering with different gas flows of N_2_ and Ar to form the top electrode. TiN is sputtered by co-sputtering pure Ti (6” × 6 mm) with N_2_. Table 1 summarizes the detailed processing parameters for the TiN electrodes. After the aforementioned processes, a post-metallization annealing was conducted through rapid thermal annealing in ambient N_2_ at 600 °C for 30 s to crystallize HZO films.

## 3. Results

**Figure 2:**
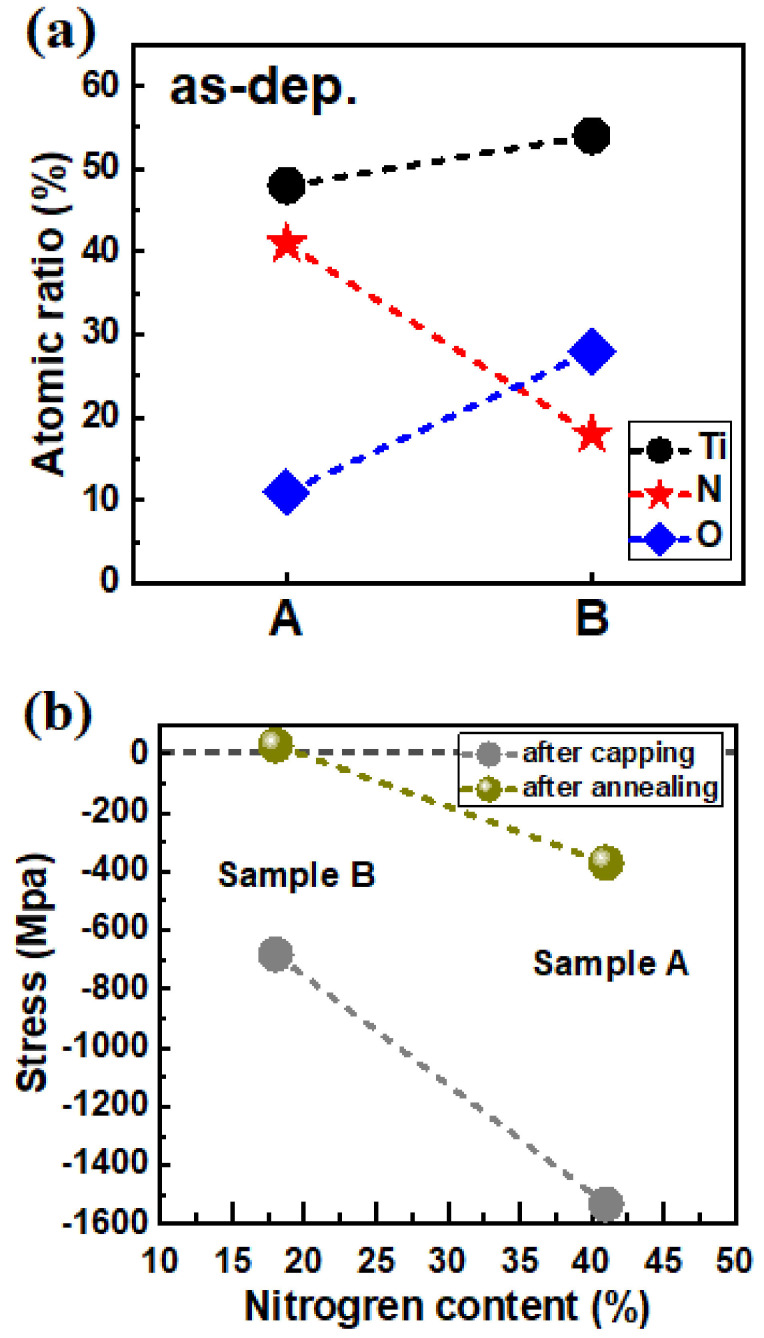
(**a**) Atomic ratio of the TiN electrodes in Sample A and Sample B and (**b**) stress as a function of the nitrogen content.

Figure 2 shows the X-ray photoelectron spectroscopy (XPS) analysis of the atomic ratio of TiN in Sample A and Sample B. The N percentage decreased from 41% to 18% as the N_2_-to-Ar gas flow ratio decreased during physical vapor deposition (PVD (Figure 2a)), and the oxygen percentage increased to compensate for the decrease in N_2_. Since the HZO is relatively thin compared with the bottom electrode and top electrode, the reporting literature only consider the coefficient of thermal expansion (CTE) between the bottom electrode and top electrode [6]. A similar approach is applied into our case since the thickness of HZO (5 nm) and SiO_2_ (1 nm) are relatively thin compared with the bottom (Si-substrate (>750μm)) and top electrode (80 nm TiN). Furthermore, we assume that the stress in HZO will be uniform through its thickness and equal to the stress in the top electrode but with an opposite direction. Therefore, we use the Stoney’s equation to estimate the stress between the TiN (top electrode) and bottom substrate (Si). The stress in the TiN film can be measured through the bending of a full wafer and calculated using Stoney’s equation (Figure 2b) [9]. Based on Stoney’s equation, a positive stress value (σ > 0) indicates the existence of in-plane compressive stress in the deposited film and in-plane tensile stress in the substrate. In our case, the HZO thin film is located below the TiN layer, leading to stress in the opposite direction to the direction of the stress acting on the TiN layer. Thus, a positive stress value (σ > 0) indicates the existence of in-plane compressive stress in the TiN layer and in-plane tensile stress in the HZO thin film. After annealing, the stress value (σ) shifts positively (i.e., from −1532.0 to −370.5 MPa for Sample A and from −681.6 to 29.8 MPa for Sample B) due to the positive CTE of TiN (i.e., 6.5 × 10^−6^/K) [6]. Furthermore, Sample B exhibits a compressive stress (29.8 MPa) characteristic, indicating that the HZO film below the TiN layer subjected to compressive strain is under tensile stress in this sample. On the other hand, Sample A exhibited tensile stress (−370.5 MPa), indicating that HZO is under compressive stress in this sample.

**Figure 3:**
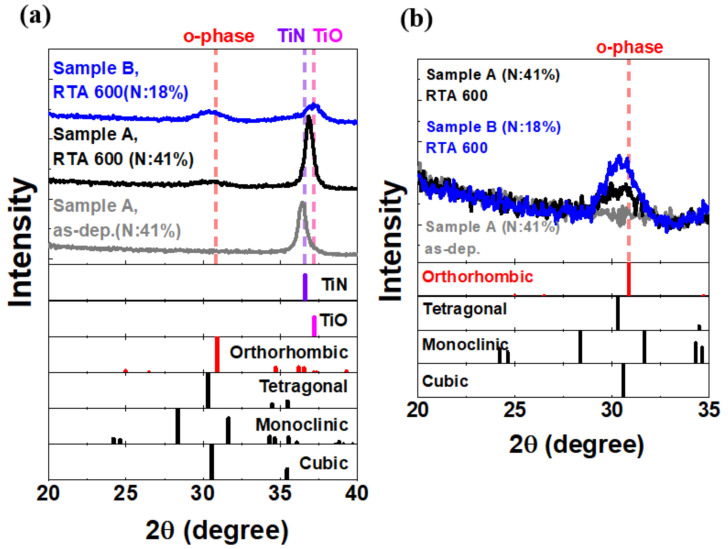
GIXRD analyses at (**a**) 20° to 40° and (**b**) almost 30° for Sample A, Sample B, and the as-dep sample.

Figure 3 shows the grazing incidence X-ray diffraction (GIXRD) analysis of Sample A, Sample B, and an as-deposited (as-dep) sample. A positive peak shift of TiN is observed by comparing the as-dep sample and Sample A, indicating that the decreased distance of the (111) plane is due to an extra compressive stress in the (111) direction after thermal annealing. Moreover, Sample B with 18% N in TiN shows an apparent TiOx peak at ~37.2°, specifying the formation of titanium oxide compounds. The higher oxygen content in the TiN (Sample B) could retard the oxygen inter-diffusion, alleviating the formation of oxygen vacancies in the HZO [10]. The coefficient of thermal expansion (CTE) of TiO_x_, i.e., 9.9 × 10^−6^/K, is considerably higher than that of TiN [11]. Therefore, the TiN with 18% N has a high compressive stress. The crystallization property of the HZO film is displayed in Figure 3b. Sample B exhibited strong GIXRD intensity in the mixed orthorhombic/tetragonal/cubic phases.

**Figure 4:**
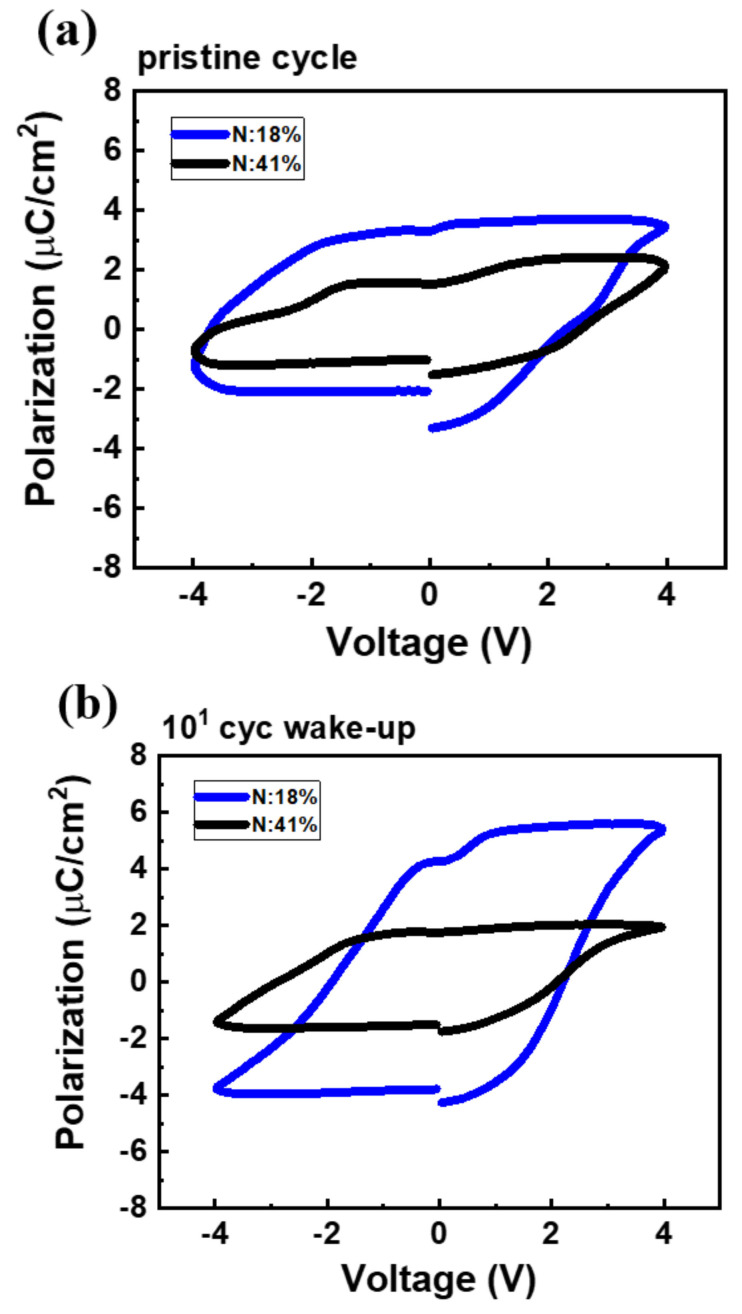
P–V characteristics of the MFIS devices on an n^+^ substrate (**a**) at the pristine state and (**b**) after 10^1^-cycle wake-up process.

For further understanding the effect of strained TiN on ferroelectricity, a positive-up- negative-down (PUND) pulsing scheme with time steps of 5 µs was used for polarization extraction. The P–V characteristics in a pristine cycle and after 10 cycles are shown in Figure 4. The TiN sample with 18% N exhibited higher 2Pr values in the pristine cycle (5.4 µC/cm^2^) and after 10 cycles (8 µC/cm^2^, which is 2.5 times the corresponding value for the sample with 41% N) than the TiN sample with 41% N.

**Figure 5:**
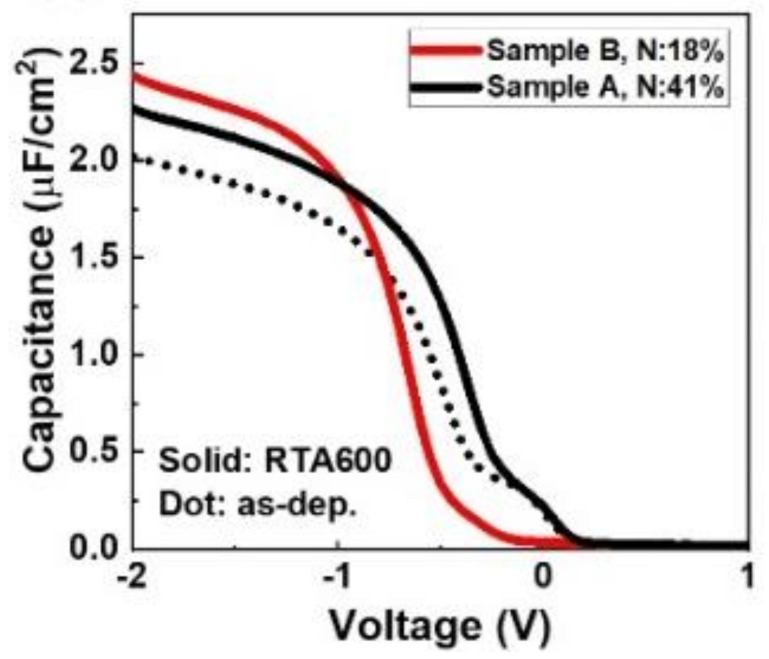
Capacitance–voltage characteristics of the pristine MFIS devices on a p substrate.

Moreover, the device with compressive strain in TiN exhibits a higher capacitance value in the accumulation region (Figure 5) than the as-dep device. This result is mainly attributed to the higher k value resulting from the orthorhombic phase than from the other phases. Furthermore, Sample B exhibits a higher capacitance than Sample A.

**Figure 6:**
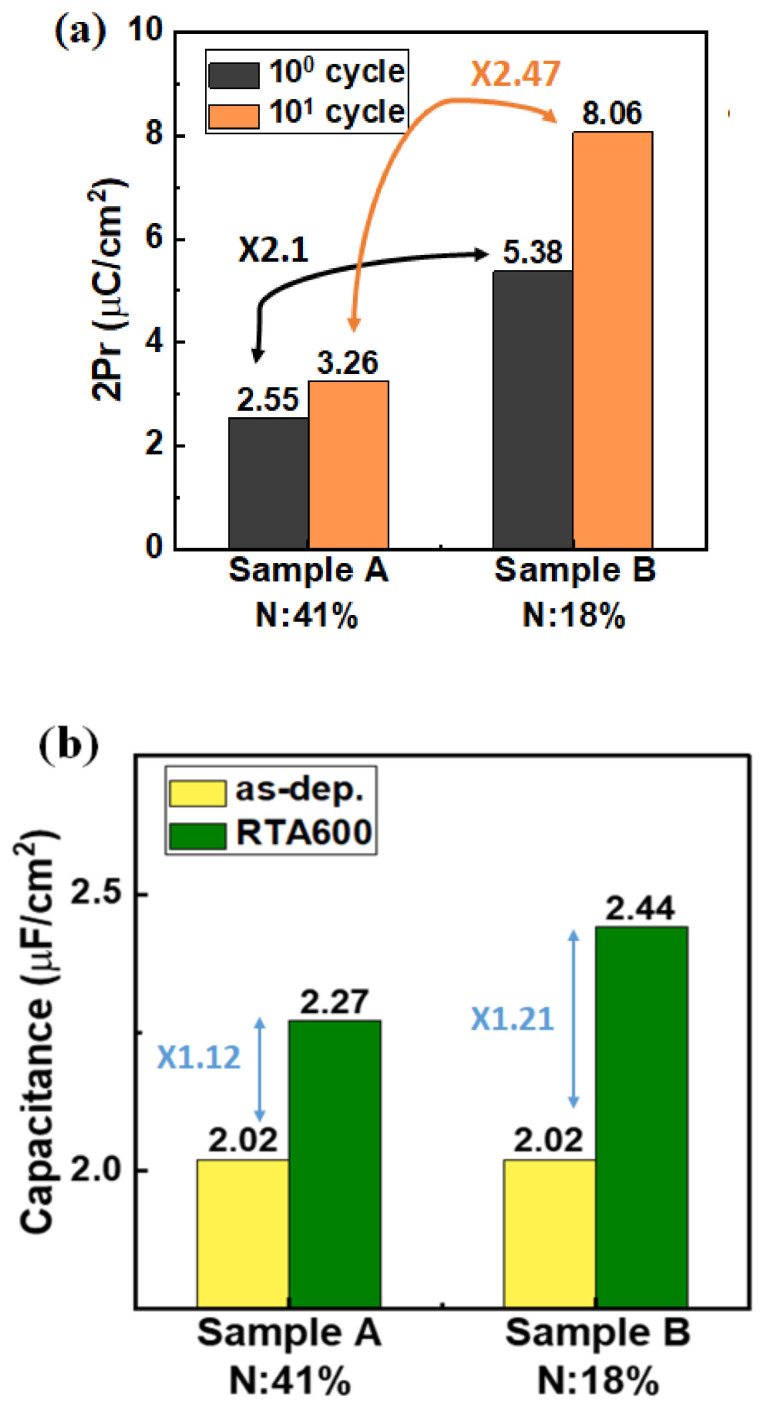
Summary of (**a**) 2Pr and (**b**) the capacitance in the accumulation region.

Figure 6 summarizes the 2Pr and capacitance values in the accumulation region in Samples A and B. An obvious increase in 2Pr and capacitance value is observed in Sample B rather than in Sample A, indicating that the tensile stress induced in the HZO film by compressive TiN with 18% N can enhance ferroelectricity.

**Figure 7:**
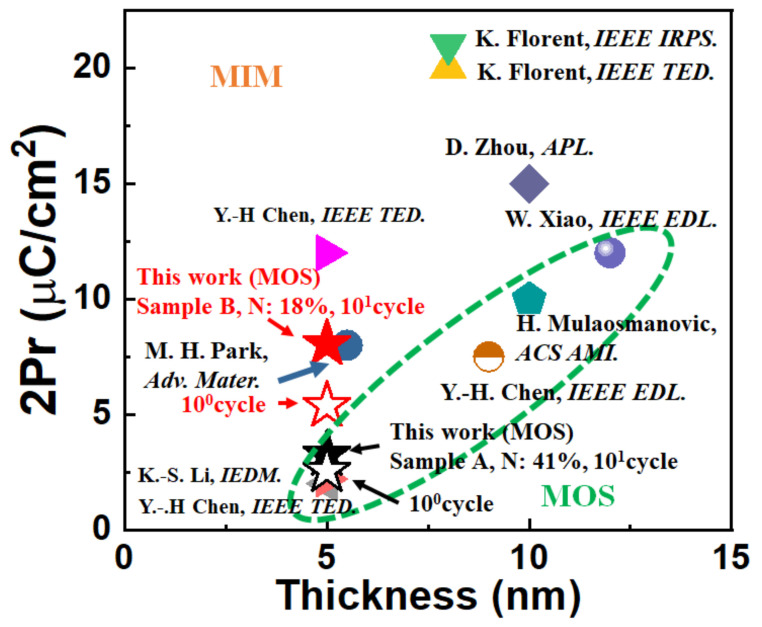
Benchmark of 2Pr for HfO_2_-based ferroelectric capacitors with different structures and film thicknesses [12,13,14,15,16,17,18,19,20].

Figure 7 shows the benchmark of 2Pr in the samples compared with other works, indicating that Sample B exhibits higher 2Pr values compared with the previous results in 5 nm HZO MFIS devices. Furthermore, Table 2 summarizes comparisons between the samples in this work and samples reported in the literature that were fabricated with different stressors. Table 2 indicates that strained TiN is a promising alternative for enhancing the ferroelectricity of sub-5 nm HZO devices.

## 4. Conclusions

In summary, we experimentally demonstrate for the first time that strained TiN can enhance ferroelectricity in 5 nm MFIS devices. First of all, with adjustment of the N content in TiN during the sputtering, the TiN with 18% N exhibited a compressive stress characteristic, and TiN with 41% N exhibited a tensile stress characteristic. Therefore, the HZO film located below the TiN with compressive strain is under tensile stress (Sample B) and HZO located below the TiN with tensile strain is under compressive stress (Sample A). Secondly, the GIXRD results indicate that the device with 18% N of TiN (Sample B) shows a stronger GIXRD intensity in the mixed orthorhombic/tetragonal/cubic phases. Finally, the device with 18% N of TiN (Sample B) shows a 2.5 times higher 2Pr value, and a larger capacitance value than the TiN device with 41% N of TiN (Sample A). Therefore, the ability of compressive TiN to boost the ferroelectricity in 5 nm HZO devices has been successfully demonstrated, indicating that the inducement of strain in TiN through the adjustment of the N content is a promising approach for enhancing ferroelectricity in sub-5 nm HZO devices.

## Figures and Tables

**Table 1:** Summary of the processing parameters for the TiN electrode.

	N_2_	Ar	DC Power	Pressure
Sample A	30 (sccm)	30 (sccm)	5000 (w)	3 (mtorr)
Sample B	1 (sccm)	100 (sccm)	800 (w)	5 (mtorr)

**Table 2:** Comparison of the devices reported in the literature and this work.

	Ref [4]			Ref [5]				Ref [6]					This work	
Structure	MFM			MFM	MFS	MFS	MFS	MFM					MFIS	
Ferroelectric layer	10 nm HZO			8 nm HZO				10 nm HZO					5 nm HZO	
Stressor	Different thickness of top electrodes (TiN)			Different materials of bottom electrodes				Different materials of top electrodes					Different N content in TiN	
Parameter	45 nm	90 nm	180 nm	TiN	Si	SiGe	Ge	Au	Pt	TiN	Ta	W	18% N in TiN	41% N in TiN
2Pr (μC/cm^2^)	35	52	48	32	none	24	36	23	32	36	36	38	8	3.26

## Data Availability

The data presented in this study are available on request from the corresponding author. The data are not publicly available due to privacy.
